# Progress in Rubella and Congenital Rubella Syndrome Control and Elimination — Worldwide, 2000**–**2016

**DOI:** 10.15585/mmwr.mm6645a4

**Published:** 2017-11-17

**Authors:** Gavin B. Grant, Susan E. Reef, Minal Patel, Jennifer K. Knapp, Alya Dabbagh

**Affiliations:** ^1^Global Immunization Division, Center for Global Health, CDC; ^2^Department of Immunization, Vaccines, and Biologicals, World Health Organization, Geneva, Switzerland.

Although rubella virus infection usually causes a mild fever and rash illness in children and adults, infection during pregnancy, especially during the first trimester, can result in miscarriage, fetal death, stillbirth, or infants with a constellation of congenital malformations known as congenital rubella syndrome (CRS) (*1*). Rubella is a leading vaccine-preventable cause of birth defects. Preventing these adverse pregnancy outcomes is the focus of rubella vaccination programs. In 2011, the World Health Organization (WHO) updated guidance on the preferred strategy for introduction of rubella-containing vaccine (RCV) into national immunization schedules and recommended an initial vaccination campaign, usually targeting children aged 9 months–14 years (*1*). The *Global Vaccine Action Plan 2011–2020* (GVAP), endorsed by the World Health Assembly in 2012, includes goals to eliminate rubella in at least five of the six WHO regions by 2020 (*2*). This report updates a previous report (*3*) and summarizes global progress toward rubella and CRS control and elimination from 2000 to 2016. As of December 2016, 152 (78%) of 194 countries had introduced RCV into the national immunization schedule, representing an increase of 53 countries since 2000, including 20 countries that introduced RCV after 2012. 

Reported rubella cases declined 97%, from 2000 (670,894 cases in 102 countries) to 2016 (22,361 cases in 165 countries). The Region of the Americas has achieved rubella and CRS elimination (verified in 2015). Rubella and CRS elimination goals have been set by the European Region (target date: 2015) and Western Pacific Region (target date to be determined), whereas the South-East Asia Region has a rubella and CRS control target. Neither the African Region nor the Eastern Mediterranean Region has set regional rubella goals or targets. To achieve the 2020 GVAP rubella elimination goals, RCV introduction needs to continue when country criteria indicating readiness for introduction are met, and rubella and CRS surveillance needs to be strengthened to ensure that progress toward elimination targets are measured. Because rubella cases are detected through measles surveillance, and because rubella vaccine is usually delivered as a combined measles-rubella vaccine, elimination activities for both diseases are programmatically linked, and measles elimination activities can be leveraged to support rubella elimination.

Rubella and CRS surveillance are necessary to assess disease burden before RCV introduction, to monitor disease burden and epidemiology after introduction, to identify pregnant women infected with rubella virus who require follow-up to assess pregnancy outcomes, and to identify, diagnose, and manage CRS-affected infants. Countries report information on immunization schedules, vaccination campaigns, number of vaccine doses administered through routine immunization services, and other WHO monitoring data (*4*) to WHO and the United Nations Children's Fund (UNICEF) each year using the Joint Reporting Form (JRF). Surveillance data, including number of cases of rubella and CRS, are also reported to WHO and UNICEF through the JRF using standard case definitions (*5*). For this report, JRF data from the period 2000–2016 were analyzed; analyses focused on data from 2000 (initiation of accelerated measles control activities), 2012 (the new phase of rubella elimination), 2014 (the last worldwide update), and 2016 (the most recent data available).

## Immunization Activities

Global coverage with RCV increased from 21% in 2000 to 40% in 2012 and to 47% in 2016. In 2000, just over half (99, 51%) of countries had introduced RCV into their immunization schedule; by the end of 2012, more than two thirds (132, 68%) of countries were using RCV. By 2014, at the time of the last worldwide update (*3*), eight additional countries introduced RCV, bringing the total number of countries using RCV to 140 (72%). At that time, 44 of the 54 countries that had not yet introduced RCV were eligible for support from Gavi, the Vaccine Alliance (Gavi).* During 2015–2016, 12 of these 54 countries introduced RCV, so that by the end of 2016, RCV had been introduced into the routine immunization schedule in 152 (78%) countries, including 13 (28%) in the African Region, 16 (76%) in the Eastern Mediterranean Region, eight (73%) in the South-East Asia Region, and all 115 countries in the Region of the Americas, European Region, and Western Pacific Region (Table 1). Among the 12 countries that introduced RCV during 2015–2016, six received Gavi support for the introduction, and six (among the 10 countries not eligible for Gavi support) introduced the vaccine using other support (Figure) (Table 2).

**TABLE 1:** Global progress in rubella and congenital rubella syndrome (CRS) control and elimination — World Health Organization (WHO) Regions, 2000, 2012, and 2016

Characteristic	WHO region (No. of countries)						
	AFR (47)	AMR (35)	EMR (21)	EUR (53)	SEAR (11)	WPR (27)	Worldwide (194)
**Regional rubella/CRS target**	**None**	**Elimination**	**None**	**Elimination**	**Control**	**Elimination**	**None**
No. of countries with RCV in schedule							
2000	2	31	12	40	2	12	**99**
2012	3	35	14	53	5	22	**132**
2016	13	35	16	53	8	27	**152**
**Regional rubella vaccination coverage (%)**							
2000	0	85	23	60	3	11	**21**
2012	0	94	38	95	5	86	**40**
2016	13	92	46	93	15	96	**47**
**No. of countries reporting rubella cases**							
2000	7	25	11	41	3	15	**102**
2012	41	35	19	47	11	23	**176**
2016	44	30	18	45	11	17	**165**
**No. of reported rubella cases**							
2000	865	39,228	3,122	621,039	1,165	5,475	**670,894**
2012	10,850	15	1,681	30,579	6,877	44,275	**94,277**
2016	4,157	1	2,037	359	10,361	5,446	**22,361**
**No. of countries reporting CRS cases**							
2000	3	18	6	34	2	12	**75**
2012	20	35	9	43	6	17	**130**
2016	21	30	10	42	10	12	**125**
**No. of reported CRS cases**							
2000	0	80	0	47	26	3	**156**
2012	69	3	20	62	14	134	**302**
2016	14	0	9	6	319	19	**367**

**Abbreviations:** AFR = African Region; AMR = Region of the Americas; CRS = congenital rubella syndrome; EMR = Eastern Mediterranean Region; EUR = European Region; RCV = rubella-containing vaccine; SEAR = South-East Asia Region; WPR = Western Pacific Region.

**FIGURE:**
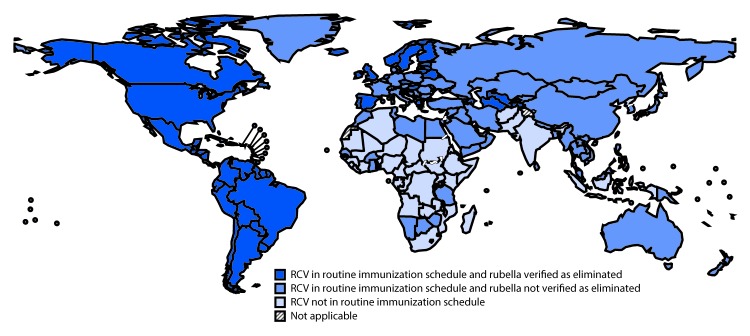
Rubella-containing vaccine (RCV) introduction and status of rubella elimination,* by country — World Health Organization, 2016 * Only the European Region and the Region of the Americas had established a process for verifying rubella elimination by July 2017.

**TABLE 2:** Characteristics of rubella-containing vaccine introduction by 12 countries that introduced the vaccine during 2015–2016, by characteristics of the introductory campaign — World Health Organization (WHO)

Country	WHO region	Year RCV introduced into routine schedule*	Introductory vaccination campaign*					Gavi support status for introduction
			Year	Target age group	Target population	% vaccination coverage by report	% vaccination coverage by survey	
Botswana	AFR	2016	2016	9 mos–14 yrs	706,504	95	97	No
Burkina Faso	AFR	2015	2014	9 mos–14 yrs	8,481,625	106†	Not reported	Yes
Burma	SEAR	2015	2015	9mos–14 yrs	13,160,764	94	Not done	Yes
Namibia	AFR	2016	2016	9 mos–39 yrs	1,859,857	103†	Not done	No
Papua New Guinea	WPR	2015	2015–2016	9 mos–14 yrs	1,976,335	63	Not done	Yes
Sao Tome and Principe	AFR	2016	2016	9 mos–14 yrs	72,449	107†	Not done	No
Swaziland	AFR	2016	2016	9 mos–14 yrs	412,874	90	94	No
Timor-Leste	SEAR	2016	2015	6 mos–14 yrs	501,832	97	95	No
Vanuatu	WPR	2015	2015	1–14 yrs	103,676	98	Not done	No
Vietnam	WPR	2015	2014–2015	1–14 yrs	19,740,181	98	Not done	Yes
Yemen	EMR	2015	2014	9 mos–14 yrs	11,368,968	85	Not done	Yes
Zimbabwe	AFR	2015	2015	9 mos–14 yrs	5,203,976	103†	Not done	Yes

**Abbreviations:** AFR = African Region; EMR = Eastern Mediterranean Region; Gavi = Gavi, the Vaccine Alliance; RCV = Rubella-containing vaccine; SEAR = South-East Asia Region; WHO = World Health Organization; WPR = Western Pacific Region.

*Introductory campaigns and introduction of the vaccine into the routine schedule can occur in different years, with introduction recommended to occur immediately following the campaign.

^†^ Values >100% indicate that the intervention reached more persons than the estimated target population.

Routine administration of RCV is recommended with the first routine dose of measles-containing vaccine (MCV1) (i.e., as a combination vaccine or simultaneously, at the same visit); this recommendation has been implemented in 144 (95%) of the 152 countries that have introduced the vaccine. Based on individual countries’ MCV vaccination schedules, the first RCV dose is scheduled at age 8–11 months in 27 (18%) countries and at age 12–18 months in 125 (83%) countries. RCV is provided as a combination vaccine with measles vaccine in 30 (20%) countries and combined with measles and mumps vaccine (with or without varicella vaccine) in 122 (80%) countries; one country administers rubella vaccine simultaneously with combined measles and mumps vaccine.

## Surveillance Activities

During 2000–2016, the number of countries reporting rubella cases (including those reporting zero cases) increased 42%, from 102 in 2000 to 176 in 2012, but the number of reporting countries declined 6%, to 165 in 2016 (Table 1). The number of countries reporting CRS cases increased 42%, from 2000 (75 countries) to 2012 (130), then decreased 4% to 125 countries in 2016. The number of reported CRS cases reported increased, especially in the South-East Asia Region, with the establishment of CRS surveillance systems. Among all 152 countries where RCV had been introduced by December 2016, 126 (83%) reported rubella data, and 110 (72%) reported CRS data.

In 2016, 22,361 rubella cases were reported to WHO, a 97% decrease from 670,894 cases reported in 2000, and a 76% decrease from 94,277 cases reported in 2012 (Table 1). Two regions (Region of the Americas and European Region) have regional verification commissions to verify rubella elimination. In the Region of the Americas, the last endemic rubella and CRS cases were reported in 2009, and the region was verified free of endemic rubella virus transmission in April 2015 (*6*). In the European Region, 33 (62%) of 53 countries were declared free of endemic rubella virus transmission in 2016.

The number of rubella virus genotype sequences identified globally from reported rubella cases increased from 33 sequences submitted by six countries in 2000, to 137 sequences submitted by 21 countries in 2012, to 188 sequences submitted by 16 countries in 2016. Of the 13 known genotypes of rubella virus, three genotypes were detected circulating in 2016.

## Discussion

In 2011, a new phase of accelerated rubella control and CRS prevention began, with updated WHO guidance for RCV introduction, Gavi funding for RCV introduction in eligible countries, and establishment of rubella elimination goals in the GVAP. Taking advantage of these opportunities and leveraging measles elimination activities, RCV has been introduced into the national immunization schedules in 53 countries since 2000; 20 (37%) of these countries introduced the vaccine during 2013–2016. By the end of 2016, with technical and financial support from partners, 78% of all countries globally had introduced RCV into their national immunization schedules, advancing progress toward elimination. Although more than three fourths of countries have introduced RCV, because of differences in country population sizes, less than half (47%) of infants worldwide are vaccinated against rubella.

Among the 42 countries that have not yet introduced RCV, nine have not achieved >80% coverage with MCV through routine immunization services or vaccination campaigns, which is a prerequisite to ensure safe RCV introduction (*1*); therefore, these nine countries need to improve routine immunization services and vaccination campaign quality. Among countries that have achieved at least 80% MCV1 coverage and are deciding whether to introduce RCV, country-specific data on CRS burden is often requested by national advisory groups or program managers to provide justification for long-term sustainable financing of RCV. Among middle-income countries that do not receive significant donor support, the financial sustainability of inclusion of RCV in the national immunization schedule is especially important to determine before embarking on introduction. Once RCV is introduced, optimizing its use is essential to reaching regional and national rubella and CRS control or elimination targets. Among the 152 countries that have introduced RCV, the vaccine was administered with MCV1 in 144 (95%) countries, facilitating the highest possible RCV coverage. In resource-limited settings, identification of the appropriate target age groups is critical to ensure reaching rubella and measles elimination goals, beginning with an introductory RCV mass vaccination campaign.

Progress toward achieving the GVAP goal of rubella elimination in five of the six WHO regions by 2020 is not on track. To achieve this goal, the three regions with elimination targets need to interrupt transmission (European and Western Pacific regions) and maintain elimination (Region of the Americas), and two of three regions will need to establish and achieve the elimination target (African, Eastern Mediterranean, and South-East Asia regions). Challenges to achieving rubella elimination goals include civil unrest that limits vaccine delivery, transmission in older populations, vaccine hesitancy in subpopulations, and weak health care service delivery with low routine vaccination coverage (*7*).

Optimal surveillance for rubella and CRS is essential to monitor the impact of rubella vaccine introduction and to verify progress toward rubella and CRS elimination goals (*8*). This requires case-based surveillance, with all cases of febrile rash illness having serum specimens tested to determine if they are measles, rubella, or neither, as well as collecting oropharyngeal specimens to identify the rubella genotypes circulating worldwide. Outbreak investigations can identify immunity gaps, and responses can be targeted to interrupt transmission and achieve and maintain elimination. Surveillance for rubella and CRS and findings from outbreak investigations guide program managers to monitor progress, focus resources to address gaps, and document elimination.

The findings in this report are subject to at least one limitation. The quality of surveillance for rubella is suboptimal. Although rubella and measles surveillance are integrated, rubella generally is a milder disease than measles, and infection is subclinical in 30%–50% of cases (*1*); therefore surveillance is much less likely to detect rubella than measles. Despite use of standard case definitions, surveillance quality varies among countries, limiting comparisons of surveillance data. Because integrated surveillance for measles and rubella is less sensitive for rubella, surveillance for CRS serves to complement the data to improve the monitoring of rubella disease.

The increase in the number of countries introducing RCV into national immunization schedules and eliminating endemic rubella virus transmission and the achievement of rubella elimination in the Region of the Americas, demonstrate progress toward global rubella control and elimination goals. Rubella and measles elimination efforts are synergistic; for example, RCV introduction catch-up campaigns, using a combined measles-rubella vaccine, also address measles immunity gaps. The path forward to reach regional rubella elimination goals is highlighted in recommendations from the Measles and Rubella Global Strategic Plan 2012–2020 Midterm Review (*7*) and requires continued improvement of routine immunization services, vaccination campaign quality, and rubella and CRS surveillance.

**Summary**

**What is already known about this topic?**

Rubella virus infection is a leading vaccine-preventable cause of birth defects. In 2011, the World Health Organization (WHO) updated guidance on the preferred strategy for introduction of rubella-containing vaccine into national routine immunization schedules, including an initial vaccination campaign for children aged 9 months–14 years. Global immunization partners have set targets to eliminate rubella and congenital rubella syndrome in at least five of the six WHO regions by 2020.

**What is added by this report?**

During 2000–2106, rubella-containing vaccine was introduced in 53 countries, including 20 introductions after 2012. By December 2016, 152 (78%) of 194 countries were using the vaccine. These introductions and increased rubella vaccine coverage globally resulted in a decrease in reported rubella cases from 670,894 cases in 2000, to 94,277 cases in 2012, to 22,361 cases in 2016. Elimination of rubella and congenital rubella syndrome was verified in the WHO Region of the Americas in 2015, and 33 (62%) of 53 countries in the European Region have now eliminated endemic rubella and congenital rubella syndrome.

**What are the implications for public health practice?**

To accelerate rubella elimination and control goals, a strong commitment to introduce rubella-containing vaccine and to achieve high rubella vaccination coverage in routine immunization services is needed in all countries. Countries and international partners should use the opportunity of measles elimination activities to achieve rubella elimination, through continued improvement of routine immunization services, vaccination campaign quality, and rubella and congenital rubella syndrome surveillance.

## Footnotes

* Four countries that had recently increased income levels above the support threshold applied and were accepted for Gavi immunization services support, but did not introduce RCV by the end of 2016.
